# Supplementary material for: Clinical burden of HDV in Spain: Incidence, prevalence, and associated comorbidities
Source: JHEP Rep. 2025 May 31;7(10):101471. doi: 10.1016/j.jhepr.2025.101471 (PMC12414884; doi:10.1016/j.jhepr.2025.101471)
Supplement: Multimedia component 1 [file mmc1.pdf]

Clinical burden of HDV in Spain: Incidence, prevalence, and associated comorbidities

Maria Buti, Nandita Kachru, Marvin Rock, Meritxell Ascanio, Josep Darba, Chong Kim

Table of content

Table S1……………………………………………………………………………………….2

Table S1. Baseline Patient Characteristics by Index Diagnosis and Age as a Covariate. All data are presented as n (%) unless stated otherwise. Data were analysed using McNemar test (Chi-squared test). Level of significance set at P < 0.05.

	**Pre-2015 Index Diagnosis**			**Post-2015 Index Diagnosis**		
**Comorbidity**	**HDV Infection**	**HBV Monoinfection**	***P*-value**	**HDV Infection**	**HBV Monoinfection**	***P*-value**
**< 45 years**	(n = 83)	(n = 1,294)		(n = 11)	(n = 183)	
Hypertension	2 (2.41)	28 (2.2)	0.4175	1 (9.1)	5 (2.7)	0.3952
History of smoking	18 (21.7)	331 (25.6)	> 0.9999	4 (36.4)	29 (15.9)	0.1857
HCV	7 (8.4)	39 (3.0)	**0.0027**	1 (9.1)	12 (6.6)	0.4236
HIV	11 (13.3)	42 (3.3)	**< 0.0001**	1 (9.1)	6 (3.3)	0.2249
Substance abuse	16 (19.3)	92 (7.1)	**< 0.0001**	2 (18.2)	13 (7.1)	0.3368
AAD/AUD	17 (20.5)	190 (14.5)	0.0982	2 (18.2)	27 (14.8)	0.2413
Diabetes	1 (1.2)	14 (1.1)	0.2764	0	2 (1.1)	> 0.9999
**≥ 45 years**	(n = 48)	(n = 1,149)		(n = 17)	(n = 294)	
Hypertension	1 (2.1)	25 (2.2)	0.4167	1 (5.9)	9 (3.1)	0.4063
History of smoking	11 (22.9)	293 (25.5)	> 0.9999	5 (29.4)	48 (16.3)	0.1725
HCV	4 (8.3)	34 (3.0)	**0.0028**	2 (11.8)	19 (6.5)	0.4297
HIV	7 (14.6)	37 (3.2)	**< 0.0001**	3 (17.7)	10 (3.4)	0.2268
Substance abuse	9 (18.8)	79 (6.9)	**< 0.0001**	3 (17.7)	21 (7.1)	0.3865
AAD/AUD	9 (18.8)	169 (14.7)	0.1085	3 (17.7)	42 (14.3)	0.2741
Diabetes	1 (2.1)	13 (1.1)	0.3751	1 (5.9)	3 (1.0)	> 0.9999

Note: All data are n (%).

AAD/AUD, alcohol abuse disorder/alcohol use disorder; HBV, hepatitis B virus; HCV, hepatitis C virus; HDV, hepatitis delta virus; HIV, human immunodeficiency virus.
