# Supplementary material for: Clinical burden of HDV in Spain: Incidence, prevalence, and associated comorbidities
Source: JHEP Rep. 2025 May 31;7(10):101471. doi: 10.1016/j.jhepr.2025.101471 (PMC12414884; doi:10.1016/j.jhepr.2025.101471)
Supplement: Multimedia component 2 [file mmc2.docx]

**JHEP Reports**

**CTAT methods**

Tables for a “Complete, Transparent, Accurate and Timely account” (CTAT) are now mandatory for all revised submissions. The aim is to enhance the reproducibility of methods.

- Only include the parts relevant to your study
- Refer to the CTAT in the main text as ‘Supplementary CTAT Table’
- Do not add subheadings
- Add as many rows as needed to include all information
- Only include one item per row

**If the CTAT form is not relevant to your study, please outline the reasons why:**

|  |
| --- |

- 1. **Antibodies - Not applicable**

| **Name** | **Citation** | **Supplier** | **Cat no.** | **Clone no.** |
| --- | --- | --- | --- | --- |
|  |  |  |  |  |

- 1. **Cell lines - Not applicable**

| **Name** | **Citation** | **Supplier** | **Cat no.** | **Passage no.** | **Authentication test method** |
| --- | --- | --- | --- | --- | --- |
|  |  |  |  |  |  |

- 1. **Organisms - Not applicable**

| **Name** | **Citation** | **Supplier** | **Strain** | **Sex** | **Age** | **Overall n number** |
| --- | --- | --- | --- | --- | --- | --- |
|  |  |  |  |  |  |  |

- 1. **Sequence based reagents - Not applicable**

| **Name** | **Sequence** | **Supplier** |
| --- | --- | --- |
|  |  |  |

- 1. **Biological samples - Not applicable**

| **Description** | **Source** | **Identifier** |
| --- | --- | --- |
|  |  |  |

- 1. **Deposited data**

| **Name of repository** | **Identifier** | **Link** |
| --- | --- | --- |
| Spanish National Health System’s Hospital Discharge Records Database (Conjunto Mínimo Básico de Datos) | CMBD | https://www.sanidad.gob.es/en/estadEstudios/estadisticas/cmbdhome.htm |

- 1. **Software**

| **Software name** | **Manufacturer** | **Version** |
| --- | --- | --- |
| Stata software | StataCorp LLC | Stata 17/18 |

- 1. **Other (*e.g*. drugs, proteins, vectors etc.) – Not applicable**

|  |  |  |
| --- | --- | --- |
|  |  |  |

- 1. **Please provide the details of the corresponding methods author for the manuscript:**

| 1. Meritxell Ascanio, BCN Health Economics & Outcomes Research SL, Barcelona, Spain 2. Chong Kim, HEOR—Global Value and Access, Gilead Sciences, Inc., Foster City, CA, USA |
| --- |

**2.0 Please confirm for randomised controlled trials all versions of the clinical protocol are included in the submission. These will be published online as supplementary information.**

| **Not applicable** |
| --- |
