# Supplementary material for: Clinical burden of HDV in Spain: Incidence, prevalence, and associated comorbidities
Source: JHEP Rep. 2025 May 31;7(10):101471. doi: 10.1016/j.jhepr.2025.101471 (PMC12414884; doi:10.1016/j.jhepr.2025.101471)
Supplement: Multimedia component 4 [file mmc4.pdf]

JHEP|Reports

Clinical burden of HDV in Spain: Incidence, prevalence, and associated comorbidities

Authors

Maria Buti, Nandita Kachru, Marvin Rock, Meritxell Ascanio, Josep Darba, Chong Kim

Correspondence

mbuti@vhebron.net (M. Buti).

Graphical abstract

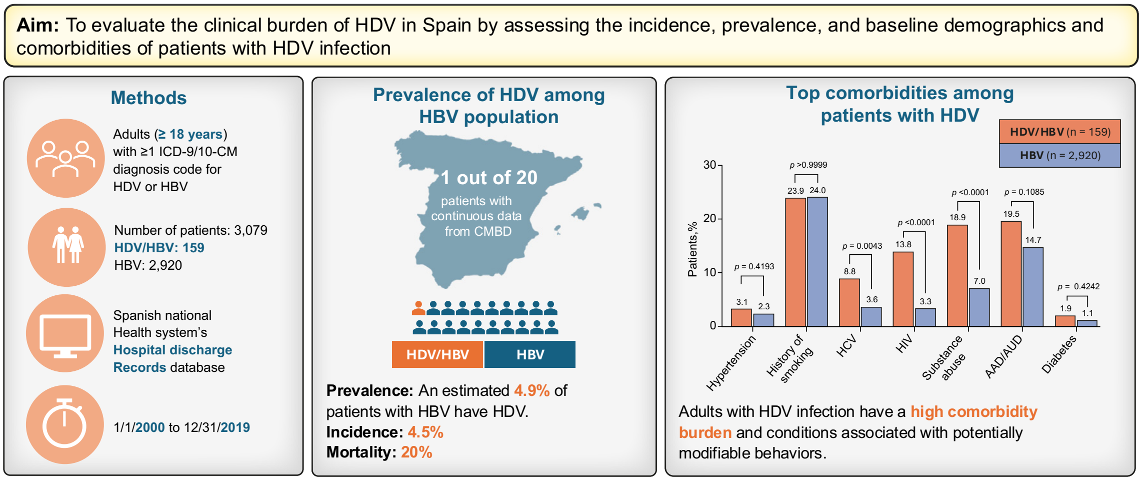

Highlights:

- HDV leads to the most severe form of viral hepatitis.
- The estimated prevalence and incidence of HDV infection in Spain were 4.9% and 4.5%, respectively.
- Adults with HDV have a high comorbidity burden.
- HDV is associated with a higher prevalence of HCV, HIV, and substance use disorder.
- The mortality rate in adults with HDV infection was 40%.

Impacts and implications:

Adults with HDV infection who attended hospitals in Spain had a high comorbidity burden and conditions associated with potentially modifiable behaviors (*i.e.* sexually transmitted diseases and substance abuse). Given the high prevalence of HDV in Spain, these comorbid conditions may contribute to a larger healthcare burden. Chronic HDV infection is also considered the most severe form of viral hepatitis, with a reported mortality rate of 40% in adults with HDV infection. These findings emphasize the importance of enhanced HDV screening in patients with chronic HBV, along with early diagnosis and the prompt initiation of antiviral therapies to manage disease progression and reduce the risk of liver-related morbidity and mortality.

https://doi.org/10.1016/j.jhepr.2025.101471
© 2025 The Authors. Published by Elsevier B.V. on behalf of European Association for the Study of the Liver (EASL). This is an open access article under the CC BY license (http://creativecommons.org/licenses/by/4.0/).

Clinical burden of HDV in Spain: Incidence, prevalence, and associated comorbidities

Maria Buti[1,*], Nandita Kachru[2], Marvin Rock[2], Meritxell Ascanio[3], Josep Darba[4], Chong Kim[2]

JHEP Reports **2025**. vol. 7 | 1–7

Check for updates

Background & Aims: HDV leads to the most severe form of viral hepatitis. It has been estimated to affect 5–13% of people who have chronic HBV worldwide. Evidence of HDV incidence, prevalence, and disease burden in Spain is limited. The purpose of this study is to evaluate the clinical burden of HDV in Spain by assessing the incidence, prevalence, and baseline demographics and comorbidities of patients with HDV infection compared with those with HBV monoinfection.

Methods: Adults (≥18 years) with ≥1 International Classification of Disease-9/10-Clinical Modification diagnosis code for HDV or HBV in the Spanish National Health System's Hospital Discharge Records Database from January 1, 2000, to December 31, 2019, were identified. HDV prevalence and incidence were calculated. Baseline (duration before index disease) patient characteristics and comorbidities were assessed.

Results: The estimated prevalence of adults with HDV infection among those with HBV was 4.9%, with an incidence of 4.5% over the study period. Adults with HDV infection were significantly younger than those with HBV monoinfection (mean [SD] age 42.7 [14.4] *vs*. 46.6 [15.9] years, p = 0.0034). Adults with HDV infection reported significantly higher rates of concomitant hepatitis C infection (8.8% *vs*. 3.6%; p = 0.0043), HIV infection (13.8% *vs*. 3.3%; p <0.0001), and substance use disorder (18.9% *vs*. 7.0%; p <0.0001) compared with those with HBV monoinfection.

Conclusions: Adults with HDV infection who attended hospitals in Spain have a high comorbidity burden and conditions associated with potentially modifiable behaviors. Novel treatment strategies are needed to reduce morbidity rates among adults with HDV infection in Spain.

© 2025 The Authors. Published by Elsevier B.V. on behalf of European Association for the Study of the Liver (EASL). This is an open access article under the CC BY license (http://creativecommons.org/licenses/by/4.0/).

Introduction

In 2019, it was estimated that 296 million people were living with chronic HBV infection worldwide, with an incidence of 1.5 million new infections each year.[1] HDV is caused by a defective RNA virus that requires the presence of HBV infection for replication and transmission.[2,3] It is the most severe form of viral hepatitis. Patients with HDV have a greater risk of cirrhosis, hepatocellular carcinoma, liver transplant, and liver-related mortality compared with patients with HBV monoinfection.[4,5] Recently, HDV was estimated to affect 5% of people who have chronic HBV (15–20 million) worldwide.[6] However, higher estimates (13%) have also been reported.[7] Regional variation exists, with the highest estimated HDV burden in South and East Asia and Africa. Prevalence rates are confounded by factors including human migratory patterns, insufficient and inconsistent screening, a general lack of surveillance, differences in access to specialists, prevalence of drug use disorder, and particularly HBV vaccination standards.[7–9]

There is a large uncertainty in HBV and HDV prevalence estimates, as many people may be unaware of their infection and diagnostic tests for HDV are not available worldwide. In 2021, the global prevalence of HBV infections was estimated to be 262 million, and approximately 1,994,000 of the HBV infections were newly diagnosed.[10] In 2021, the POLARIS Observatory Collaborators reported the global and country prevalence of HDV and estimated a lower HDV prevalence among people living with HBV in 18 out of 25 countries, as previous analyses focused on studies conducted in groups/regions that have a higher probability of HBV infection, such as tertiary care centers, specific risk groups, or geographical regions.[11] Accurate estimates are needed to formulate strategies for diagnosing coinfected individuals more effectively and efficiently, and reflex testing may be the answer. The implementation of double reflex testing was recommended as the most effective method for developing accurate estimates of the prevalence of anti-HDV and HDV RNA positivity and identifying undiagnosed individuals.[10] In addition, recent data from Spanish centers indicate that the consistent implementation of reflex testing may increase the detection rate for HDV-infected cases by 8–10 times.[12]

Although a cure for HBV or HDV does not exist yet, antiviral treatments are available to help slow the progression of the disease.[13] The EASL clinical practice guidelines on HDV recommend that all patients with chronic hepatitis D and

* Corresponding author. Address: Liver Unit, Hospital Universitario Valle Hebron, Paseo Valle Hebron 119, Barcelona 08035, Spain.
E-mail address: mbuti@yhebron.net (M. Buti).
https://doi.org/10.1016/j.jhepr.2025.101471

compensated liver disease be considered for treatment. Two drugs are available for the therapy of HDV. Bulevirtide, an entry inhibitor that prevents the infection of hepatocytes by HDV and HBV, is the only approved drug by EMA and is currently considered a first-line treatment option.[14] By contrast, pegylated interferon alpha (Peg-IFNα) is not approved but is often recommended for the treatment of HDV. However, the efficacy of Peg-IFN is low because of frequent side effects, multiple contraindications that often inhibit use in patients with cirrhosis or decompensated cirrhosis, and the association with a decreased likelihood of disease progression. There is a critical need to improve screening and monitoring for HBV and HDV to facilitate effective treatment and mitigate progression.

Despite an earlier notion that HDV prevalence may be decreasing in some European countries,[15] it has been reported that HDV infection burden still appears to be significant, particularly in migrants from countries with suboptimal HBV vaccination programs.[5] In fact, more recent data have shown that the prevalence of HDV has remained stable or has increased in many endemic and non-endemic countries because of an increase in associated risk factors.[5] For example, immigration has contributed to an increasing HDV infection rate in Greece, Italy, and Spain.[15] Recent evidence of incidence, prevalence, and disease burden of HDV in Spain is limited. The purpose of this study is to evaluate the clinical burden of HDV in Spain by assessing the incidence, prevalence, and baseline demographics and comorbidities of patients with HDV infection compared with those with HBV monoinfection.

Patients and methods

Data source

This retrospective cohort study obtained data from the Spanish National Health System's Hospital Discharge Records Database (Conjunto Mínimo Básico de Datos; https://www.sanidad.gob.es/en/estadEstudios/estadisticas/cmbdhome.htm). The database covers 192 private and 313 public hospitals with >40 million patients, which accounts for >90% of the Spanish population. Parameters such as health centers and medical history identifiers were recoded before extraction to maintain anonymous records with no access to identifying information, in accordance with the principles of Good Clinical Practice and the Declaration of Helsinki. The Spanish legislation did not require patient consent and ethics committee approval.[16]

The study period ranged from January 1, 2000, to December 31, 2019, and the patient identification period was from January 1, 2001, to December 31, 2018 (Fig. 1). The baseline period was 12 months before the index date, which was defined as the earliest date of HDV diagnosis during the subject identification period. Individuals with evidence of HDV/HBV during the baseline period (12 months before the index diagnosis date) were excluded from the study to identify incident cases. The follow-up period was 12 months after the index date.

Patient population

The patient population included adults ≥18 years of age with ≥1 International Classification of Disease (ICD)-9/10-Clinical Modification (CM) diagnosis code for HDV or HBV in the database. Patients with prior HDV or prior HBV were excluded.

Baseline characteristics and outcomes

Baseline demographics and clinical characteristics were assessed over the entire duration before the index diagnosis date. Baseline demographics included age, sex, physician specialty, payer channel, and geographic region. Age at index date was retained in the dataset as a continuous variable and stratified into the following age groups: 18–34, 35–44, 45–54, 55–64, 65–74, and ≥75 years. Geographic region at index date was determined by the postal code of the indexing provider, and states were categorized into six health plan regions: Andalusia (south), Catalonia (northeast), Galicia (northwest), Madrid (central), Valencian Community (southeast), and other. Primary insurance payer at index date was classified as public (social security) or private.

Clinical characteristics and comorbidities (*i.e.* history of smoking, alcohol abuse disorder [AAD]/alcohol use disorder [AUD], substance abuse, HCV infection, HIV infection, hypertension, and diabetes) were captured at baseline using ICD-9/10-CM codes. In addition, the Quan-Charlson Comorbidity Index (CCI) score was used to assign a weighted score from 1 to 6 to evaluate patients' comorbidity during the baseline period (12 months before the index date).

The outcomes described included patient baseline clinical characteristics, prevalence, and incidence of HDV. Prevalence was measured as the proportion of people with HDV among those with HBV. Incidence was the proportion of people with new diagnoses of HDV infection among the at-risk population.

Statistical analysis

Demographics and patient comorbidities were summarized and are reported at baseline. The mean (SD), median (IQR), minimum, and maximum values are reported for all continuous variables. Descriptive statistics such as counts (frequencies) and percentages are reported for categorical values. Wilcoxon signed-rank tests were used to compare all continuous measures, and McNemar tests were used to compare dichotomous variables.

All analyses were performed using Stata software (StataCorp LLC, College Station, Texas, United States), and two-tailed statistical significance was determined *a priori* at $p < 0.05$.

Results

Study population

A total of 12,317 different or consecutive patients with a diagnosis of HDV infection or HBV monoinfection between January 1, 2001, and December 31, 2018, were identified in the

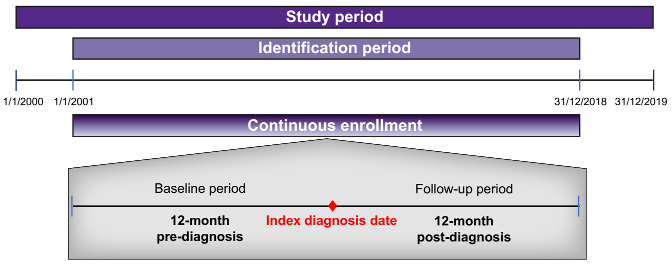

Fig. 1. Study design.

Spanish National Health System's Hospital Discharge Records Database (Conjunto Mínimo Básico de Datos). Of these patients, 11,939 met the criteria for age (≥18 years), with 582 patients diagnosed with an HDV infection and 11,357 patients determined to have an HBV monoinfection. A total of 3,079 patients (HDV infection, n = 159; HBV monoinfection, n = 2,920) met the inclusion criteria for continuous enrollment (Fig. 2).

Prevalence and incidence

Prevalence was calculated as the proportion of patients diagnosed with HDV infection identified in the claims data (n = 582) among the total number of patients diagnosed with HBV monoinfection and/or HDV infection (n = 11,939). The estimated prevalence of adults with HDV among those with HBV was 4.9% over the study period.

Incidence was calculated as the proportion of new HDV diagnoses during the study period (n = 536) among the total at-risk population (total number of patients diagnosed with HBV monoinfection and/or HDV infection; n = 11,939). The estimated incidence rate of adults with HDV among those with HBV was 4.5% over the study period.

Patient and clinical characteristics

Table 1 reports the patient and clinical characteristics for individuals diagnosed with either HDV infection or HBV monoinfection during the study period.

Patients with HDV infection were significantly younger than those with HBV monoinfection (mean [SD] age: 42.7 [14.4] *vs.* 46.6 [15.9] years; p = 0.0034; Table 1). A higher proportion of patients aged 65–74 and >75 years was observed in the HBV monoinfection group. A higher proportion of males was captured across both groups, compared with women (males: ~75%; women: ~25%). No statistically significant difference was detected in mean (SD) Charlson Comorbidity Index (CCI) score between adults diagnosed with HDV infection and those diagnosed with HBV monoinfection (0.9 [1.8] *vs.* 0.8 [1.3]). The majority of individuals were covered by public insurance, and nearly half were seen by gastroenterologists.

Baseline comorbidities

Adults with HDV infection reported significantly higher rates of comorbid HCV infection (14 [8.8%] *vs.* 104 [3.6%]; p = 0.0043), HIV infection (22 [13.8%] *vs.* 95 [3.3%]; p < 0.0001), and substance use disorder (30 [18.9%] *vs.* 205 [7.0%]; p < 0.0001) compared with those with HBV monoinfection (Fig. 3). Adults with HDV infection had numerically higher rates of hypertension (5 [3.1%] *vs.* 67 [2.3%]; p = 0.4193), AAD/AUD (31 [19.5%] *vs.* 428 [14.7%]; p = 0.1085), and diabetes (3 [1.9%] *vs.* 32 [1.1%]; p = 0.4242) compared with those with HBV monoinfection.

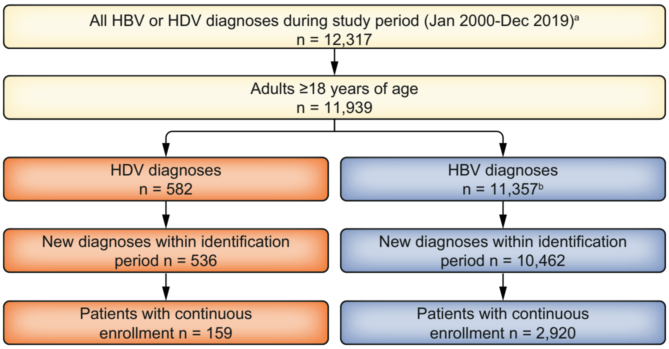

Fig. 2. Patient flowchart. a Identification period was from January 2001 to December 2018. b May include patients with HDV diagnoses. HBV, hepatitis B virus; HDV, hepatitis delta virus.

Subgroup analysis by index diagnosis: patient and clinical characteristics, and mortality

Table 2 presents the patient and clinical characteristics by index diagnosis date (pre-2015 and post-2015) for adults with HDV infection and HBV monoinfection. Compared with adults with HBV monoinfection, those with HDV infection exhibited a higher prevalence of the same comorbidities before the index diagnosis date as within the overall population: comorbid HCV infection (11 [8.4%] *vs.* 73 [3.0%]; p = 0.0032), HIV infection (18 [13.8%] *vs.* 79 [3.2%]; p <0.0001), and substance use disorder (25 [19.1%] *vs.* 171 [7.0%]; p <0.0001). However, no significant differences were observed between the HDV infection and HBV monoinfection groups after the 2015 index date.

Index diagnosis dates had no effect on mortality rates between adults with HDV infection and those with HBV monoinfection (Table 3). The mortality rate in the overall sample after the index date was the same for adults with HDV infection as for those with HBV monoinfection (40%; p = 0.2041).

Subgroup analysis by age: baseline comorbidities

Further analyses were conducted to assess whether age (<45 *vs.* ≥45 years) acted as a confounder between the comorbidity rates analyzed in the HBV monoinfection and HDV infection groups. Significant differences were observed in both the younger (<45 years) and older (≥45 years) age groups, but only when the index diagnosis occurred before 2015. In the pre-2015 cohort, younger and older adults with HDV infection reported significantly higher rates of comorbid HCV infection (p <0.003), HIV infection (p <0.0001), and substance use disorder (p <0.0001) compared with those with HBV monoinfection. The results are described in Appendix S1.

Discussion

The present study used data from a national hospital database in Spain to determine that the estimated prevalence of HDV among adults with HBV was 4.9%, with an incidence rate of 4.5% over the study period. In this dataset, adults with HDV infection were significantly younger than those with HBV monoinfection. Adults with HDV infection had a greater prevalence of HCV infection, HIV infection, and substance use disorder when compared with adults with HBV monoinfection. This result was consistent in adults with HDV infection in the pre-2015 index date subgroup, suggesting that the diagnosis date was a key factor driving the higher rate of comorbidities. This suggests adults with HDV infection may be clinically more complex for management.

To our knowledge, this is the largest study performed to date in Spain, and the reported prevalence (4.9%) at hospital levels is similar to other global and Spanish estimates of HDV prevalence.[6] In a case–control study performed between 1998 and 2012, including 429 patients with chronic HBV infection from a region in northern Spain, 6.1% were anti-HDV antibody

Table 1. Baseline patient characteristics.

Characteristic	HDV infection (n = 159)	HBV monoinfection (n = 2,920)	*p* value
Male	117 (73.6)	2,131 (73.0)	0.9270
Age (years), mean (SD)	42.7 (14.4)	46.6 (15.9)	**0.0034**
Age category (years)			
18–34	50 (31.5)	721 (24.7)	0.0602
35–44	44 (27.7)	756 (25.9)	0.6425
45–54	39 (24.5)	600 (20.6)	0.2288
55–64	11 (6.9)	407 (13.9)	0.7640
65–74	11 (6.9)	237 (8.1)	**<0.0001**
≥75	4 (2.5)	199 (6.8)	**0.0315**
Region			
Andalusia (south)	25 (15.7)	486 (16.6)	0.8273
Catalonia (northeast)	26 (16.4)	476 (16.3)	>0.9999
Galicia (northwest)	8 (5.0)	180 (6.2)	0.7329
Madrid (central)	31 (19.5)	485 (16.6)	0.3280
Valencian Community (southeast)	18 (11.3)	379 (13.0)	0.6273
Other	51 (32.1)	914 (31.3)	0.8607
Physician specialty			
Gastroenterology	71 (44.7)	1,469 (50.3)	0.1673
Internal medicine	30 (18.9)	581 (19.9)	0.8383
Other	58 (36.5)	870 (29.8)	0.0763
Payer channel			
Public	149 (93.7)	2,786 (95.4)	0.3311
Private	4 (2.5)	68 (2.3)	0.7867
Quan-CCI score, mean (SD)	0.9 (1.8)	0.8 (1.3)	0.5793
CCI group			
0	107 (67.3)	1,854 (63.5)	0.3525
1	22 (13.8)	494 (16.9)	0.3825
2	9 (5.7)	245 (8.4)	0.2982
3	6 (3.8)	159 (5.5)	0.4693
≥4	15 (9.4)	168 (5.8)	0.0815

All data are presented as n (%) unless stated otherwise. Data were analyzed using the McNemar test (Chi-squared test for dichotomous variables) or the Wilcoxon test (continuous variables). Level of significance set at p <0.05. CCI, Charlson Comorbidity Index; HBV, hepatitis B virus; HDV, hepatitis delta virus; Quan-CCI, quantitative Charlson Comorbidity Index; SD, standard deviation.

positive.[17] Multivariate logistic regression analyses identified the following factors associated with the presence of anti-HDV antibodies: immigration, injecting drug use (IDU), sexual transmission, and high alanine aminotransferase values. In a separate study that assessed data from 478 HBV patients in northwestern Spain, 19 (4%) of patients had anti-HDV antibodies detectable at the first diagnosis of HBV.[18] The prevalence of HDV infection is similar in previous studies because the analysis was mainly performed at the hospital level, whereas primary care data are scarce. The prevalence of HDV in the primary care data would most likely be lower in this setting. In our study, most patients with HDV were male, former injection drug users, and native to Spain. At the global level, a large systematic review and network meta-analysis reported a global prevalence of HBV/HDV infection among those diagnosed with HBV at 4.5%.[6] Similarly, in a large US database capturing approximately 80% of the US-insured population, HBV/HDV infection prevalence was 4.6% among adults infected with HBV.[19] The POLARIS Observatory Collaborators reported data from 25 countries and territories, which account for 37% of the global HBV-infected population.[11] After adjusting for geographical distribution, disease stage, and special populations, the anti-HDV prevalence differed from the estimates found in the literature for 19 countries. The analysis resulted in a much lower global anti-HDV prevalence of 2.0% than previously reported, as prior meta-analyses primarily focused on studies conducted in groups/regions that have a higher probability of HBV infection. Following the application of serosurvey data to the Spanish population (208,000 adults with HBsAg+ in 2023), the overall anti-HDV prevalence was calculated at 2.3% compared with the literature prevalence of 5.2%. Therefore, the prevalence of HDV reported in our assessment of multiregional patient data from Spain is similar to that reported in other global and region-specific studies of patient data from Spain.

At diagnosis, a significantly larger proportion of patients diagnosed with HDV infection presented with comorbid conditions compared with patients with HBV monoinfection. The

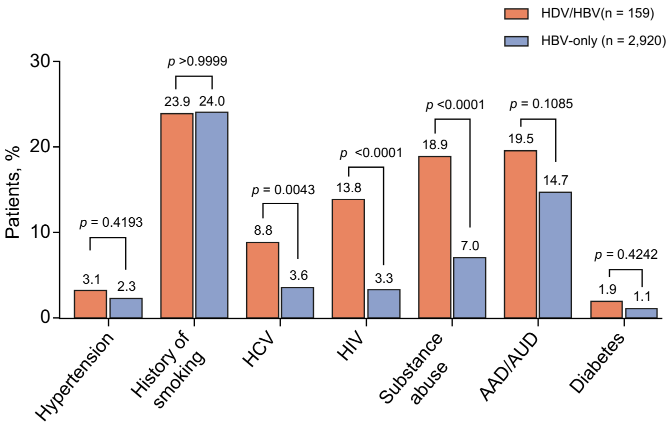

Fig. 3. Baseline comorbidities in adults with HDV infection and HBV monoinfection. Bars represent the percentage of patients. Data were analyzed using the McNemar test (Chi-squared test). Level of significance set at p <0.05. AAD, alcohol abuse disorder; AUD, alcohol use disorder; HBV, hepatitis B virus; HDV, hepatitis delta virus.

Table 2. Baseline patient characteristics by index diagnosis.

	Pre-2015 index diagnosis			Post-2015 index diagnosis		
**Characteristic**	**HDV infection (n = 131)**	**HBV monoinfection (n = 2,443)**	***p* value**	**HDV infection (n = 28)**	**HBV monoinfection (n = 477)**	***p* value**
Male	97 (74.1)	1,781 (72.9)	0.8720	20 (71.4)	350 (73.4)	0.8417
Age (years), mean (SD)	42.0 (14.4)	45.8 (15.8)	**0.0031**	46.1 (14.1)	50.7 (15.5)	0.4623
Age category (years)						
18–34	43 (32.8)	652 (26.7)	0.0637	7 (25.0)	69 (14.5)	0.8341
35–44	40 (30.5)	642 (26.3)	0.6512	4 (14.3)	114 (23.9)	0.6792
45–54	27 (20.6)	483 (1.8)	0.2308	12 (42.9)	117 (24.5)	0.7514
55–64	9 (6.9)	323 (13.2)	0.7591	2 (7.1)	84 (17.6)	0.9068
65–74	9 (6.9)	184 (7.5)	**<0.0001**	2 (7.1)	53 (11.1)	0.7163
≥75	3 (2.3)	159 (6.5)	**0.0326**	1 (3.6)	40 (8.4)	0.7096
Region						
Andalusia (south)	19 (14.5)	414 (17.0)	0.7934	6 (21.4)	72 (15.1)	0.6134
Catalonia (northeast)	21 (16.0)	376 (15.4)	>0.9999	6 (21.4)	100 (21.0)	0.3921
Galicia (northwest)	8 (6.1)	153 (6.3)	0.7268	0	27 (5.7)	>0.9999
Madrid (central)	29 (22.1)	405 (16.7)	0.3614	6 (21.4)	80 (16.8)	0.1582
Valencian Community (southeast)	17 (13.0)	321 (13.1)	0.5982	1 (3.6)	58 (12.2)	0.4843
Other	37 (28.2)	774 (31.7)	0.8531	9 (32.1)	140 (29.4)	0.3251
Physician specialty						
Gastroenterology	54 (41.2)	1,166 (47.7)	0.1493	17 (60.7)	303 (63.5)	>0.9999
Internal medicine	23 (17.6)	463 (19.0)	0.8236	7 (25.0)	118 (24.7)	0.2136
Other	54 (41.2)	814 (33.3)	0.0748	4 (14.3)	56 (11.7)	**0.0009**
Payer channel						
Public	124 (94.7)	2,327 (95.3)	0.3137	25 (89.3)	459 (96.2)	0.0957
Private	3 (2.3)	61 (2.5)	0.7682	1 (3.6)	7 (1.5)	0.6918
Quan-CCI score, mean (SD)	0.9 (1.9)	0.7 (1.3)	0.5591	0.86 (0.50)	0.79 (1.38)	**<0.0001**
CCI group						
0	91 (69.5)	1,555 (63.7)	0.3268	16 (57.1)	299 (62.7)	**0.0493**
1	15 (11.5)	494 (16.9)	0.3652	7 (25.0)	83 (17.4)	0.6872
2	6 (4.6)	245 (8.3)	0.2896	3 (10.7)	41 (8.6)	0.3945
3	6 (4.6)	159 (5.3)	0.4533	0	29 (6.1)	0.5236
≥4	13 (9.9)	168 (5.9)	0.0794	2 (7.1)	25 (5.2)	**0.0021**
Infection						
Tripe infected (total)	29 (22.1)	N/A	N/A	7 (25.0)	N/A	**0.0435**
Triple infected (HBV/HDV/HCV)	11 (8.4)	N/A	N/A	3 (10.7)	N/A	0.1872
Triple infected (HBV/HDV/HIV)	18 (13.7)	N/A	N/A	4 (14.3)	N/A	0.2948
Quadruple infected (HBV/HDV/HCV/HIV)	7 (5.3)	N/A	N/A	1 (3.6)	N/A	0.5639
Baseline comorbidities						
Hypertension	3 (2.3)	53 (2.2)	0.4038	2 (7.1)	14 (2.9)	0.4382
History of smoking	29 (22.1)	624 (25.5)	>0.9999	9 (32.1)	77 (16.1)	0.1785
HCV	11 (8.4)	73 (3.0)	**0.0032**	3 (10.7)	31 (6.5)	0.4163
HIV	18 (13.7)	79 (3.2)	**<0.0001**	4 (14.3)	16 (3.4)	0.2374
Substance abuse	25 (19.1)	171 (7.0)	**<0.0001**	5 (17.9)	34 (7.1)	0.3559
AAD/AUD	26 (19.9)	359 (14.7)	0.1172	5 (17.9)	69 (14.5)	0.2757
Diabetes	2 (1.5)	27 (1.1)	0.3946	1 (3.6)	5 (1.1)	>0.9999

All data are presented as n (%) unless stated otherwise. Data were analyzed using the McNemar test (Chi-squared test for dichotomous variables) or the Wilcoxon text (continuous variables). Level of significance set at $p < 0.05$. AAD, alcohol abuse disorder; AUD, alcohol use disorder; CCI, Charlson Comorbidity Index; HBV, hepatitis B virus; HCV, hepatitis C virus; HDV, hepatitis delta virus; HIV, human immunodeficiency virus; N/A, not applicable; Quan-CCI, quantitative Charlson Comorbidity Index; SD, standard deviation.

Table 3. Post-index date mortality rates.

	HDV infection	HBV monoinfection	*p* value
Overall sample	64 (40.3)	1,168 (40.0)	0.2041
Pre-2015 index diagnosis cohort	53 (33.3)	977 (33.5)	0.3172
Post-2015 index diagnosis cohort	11 (6.9)	191 (6.5)	0.1936

All data are presented as n (%). Data were analyzed using the McNemar test (Chi-squared test). Level of significance set at p <0.05. HBV, hepatitis B virus; HDV, hepatitis delta virus.

top conditions for patients with HDV infection were substance abuse, HIV, and HCV infection. These results may potentially help identify specific "high-risk" populations of HDV that may benefit from targeted outreach programs to improve HDV screening. However, these comorbid conditions can also lead to a larger healthcare burden. HDV has been associated with higher health care use and cost burden than HBV alone.[20] A case–control study using the Truven Health MarketScan Commercial Claims databases in the USA reported higher total annual health care costs ($19,476 *vs*. $23,605; p < 0.0001) after diagnosis in 2,727 patients with HDV compared with before diagnosis. Because of the high prevalence of HDV in Spain, there is a need to better understand the economic burden related to HDV infection.

There continues to be a lack of consistent global guidance on the effective screening, approved assays, and diagnosis of HDV. Although the EASL guidelines suggests that all adults with HBV be considered for HDV screening to exclude other potential etiologies contributing to chronic liver disease,[14] the AASLD guidelines recommend anti-HDV testing only in those HBsAg-positive adults who are at risk (*i.e.* immigrants from regions with high HBV/HDV infection endemicity, persons who have injected drugs, men who have sex with men, individuals with HCV or HIV infections, persons with multiple sex partners or history of sexually transmitted infections, and individuals with elevated alanine aminotransferase or aspartate transaminase liver enzymes with low or undetectable HBV DNA).[21] Despite these guidelines, the lack of reflex testing performed nationally results in undiagnosed HDV cases.[22] For example, recent data from centers in Spain have indicated that the rate of HDV testing in routine clinical practice is extremely low, with only 7.6% of HBsAg-positive patients being tested for anti-HDV before implementing reflex testing, leading to a large number of potential HDV cases going undetected.[12] Although the overall prevalence of anti-HDV positive cases remained similar before and after reflex testing (9.6% *vs*. 8.1%, respectively), consistent implementation of reflex testing increased the absolute number of detected HDV cases five-fold. Similarly, a Spanish modeling analysis over an 8-year time frame reported that the implementation of reflex testing would increase anti-HDV detection by 5,498 cases (582 with no reflex testing to 6,080 with reflex testing) and HDV RNA by 3,225 cases (423 with no reflex testing to 3,648 with reflex testing).[22] By 2030, the use of anti-HDV reflex testing could lead to a greater than nine-fold increase in HDV diagnoses, and a reduction of the clinical and economic burden of HDV by 35–38%.[22] There is a need for improved adherence to guidelines by routinely testing all HBsAg-positive patients for HDV to accurately diagnose and manage potential co-infections.

Chronic HDV infection is considered the most severe form of viral hepatitis and can lead to rapid liver disease progression, cirrhosis, and liver-related death.[4,5] In our study, the mortality rate in adults with HDV infection was 40%. A recent systematic literature review (SLR) evaluated the role of HDV RNA status as a risk factor for disease progression.[23] Patients with an HDV RNA+ status were at a higher risk of mortality compared with those who were HDV RNA– (four studies: hazard ratio 3.78, 95% confidence interval 2.18–6.56). These findings emphasize the importance of enhanced HDV screening in patients with chronic HBV, along with early diagnosis and the prompt initiation of antiviral therapies to manage disease progression and reduce the risk of liver-related morbidity and mortality.

The greatest strength of this study is the inclusion of a large sample of patients from a national database, thereby providing results that are generalizable to the broader population. Patient demographics, clinical characteristics, and prevalence by geographic location were captured specifically for the Spanish population, providing an evidence-based resource toward the improvement of screening in this high-risk population.

The usual limitations of retrospective claims analyses apply, as diagnoses made via ICD codes are subject to miscoding and can lead to misclassification bias. In addition, a lack of approved assays and suboptimal screening practices to determine HDV and HBV status may have resulted in an underestimation of the actual number of people with HDV infection. Lastly, the dataset contains limited information on treatment and laboratory testing, which represents a limitation. Comprehensive data in these areas would have provided deeper insights into patient severity and treatment patterns.

In summary, this comprehensive database analysis estimates a 4.9% prevalence rate of HDV infection among patients with HBV diagnosed in Spain from 2000 to 2019. Adults with HDV infection who attended hospitals in Spain have a high comorbidity burden and conditions associated with potentially modifiable behaviors (*i.e.* sexually transmitted diseases and substance abuse). Novel treatment strategies are needed to improve outcomes and reduce morbidity rates among adults with HDV infection in Spain.

Affiliations

[1]*Liver Unit, Hospital Universitario Valle Hebron, Barcelona, Spain;* [2]*HEOR—Global Value and Access, Gilead Sciences, Inc., Foster City, CA, USA;* [3]*BCN Health Economics & Outcomes Research SL, Barcelona, Spain;* [4]*Department of Economics, University of Barcelona, Barcelona, Spain*

Abbreviations

AAD, alcohol abuse disorder; AASLD, American Association for the Study of Liver Diseases; AUD, alcohol use disorder; CCI, Charlson Comorbidity Index; CM, Clinical Modification; EASL, European Association for the Study of the Liver; EMA, European Medicines Agency; HBV, hepatitis B virus; HCV, hepatitis C virus; HDV, hepatitis delta virus; HIV, human immunodeficiency virus; ICD, International Classification of Disease; IDU, injecting drug use; IQR, interquartile range; Peg-IFN, pegylated interferon; Quan-CCI, quantitative Charlson Comorbidity Index; SD, standard deviation; SLR, systematic literature review.

Financial support

This study was supported by Gilead Sciences Inc.

Conflicts of interest

MB reports receiving teaching/speaking fees, grants, and consulting fees from AbbVie, Gilead, and Janssen; **NK, MR,** and **CK** are employees of Gilead Sciences, Inc., and may own stock in Gilead Sciences, Inc.; **MA** is an employee of BCN Health Economics & Outcomes Research SL, an independent contract health economic organization; and **JD** is employed by the University of Barcelona.

Please refer to the accompanying ICMJE disclosure forms for further details.

Authors' contributions

Data collection: MB, NK, MA, JD. Statistical analysis: MA, CK. Concept, design, and writing of the article: MB, NK, CK. Review of the article: MB, NK, MR, MA, JD. Revision: CK.

Data availability statement

Data were obtained from the Spanish National Health System's Hospital Discharge Records Database (Conjunto Mínimo Básico de Datos; https://www.sanidad.gob.es/en/estadEstudios/estadisticas/cmbdhome.htm). Data are available upon reasonable request.

Acknowledgements

Medical writing and editorial support were provided by the Maple Health Group.

Supplementary data

Supplementary data to this article can be found online at https://doi.org/10.1016/j.jhepr.2025.101471.

References

[1] World Health Organization. Global progress report on HIV, viral hepatitis and sexually transmitted infections, 2021. Accountability for the global health sector strategies 2016–2021: actions for impact. https://apps.who.int/iris/bitstream/handle/10665/342813/9789240030992-eng.pdf Accessed 10 October 2021.
[2] Mentha N, Clement S, Negro F, et al. A review on hepatitis D: from virology to new therapies. J Adv Res 2019;17:3–15.
[3] Lucifora J, Delphin M. Current knowledge on hepatitis delta virus replication. Antivir Res 2020;179:104812.
[4] Bockmann JH, Grube M, Hamed V, et al. High rates of cirrhosis and severe clinical events in patients with HBV/HDV co-infection: longitudinal analysis of a German cohort. BMC Gastroenterol 2020;20:24.
[5] Da BL, Heller T, Koh C. Hepatitis D infection: from initial discovery to current investigational therapies. Gastroenterol Rep (Oxf) 2019;7:231–245.
[6] Stockdale AJ, Kreuels B, Henrion MYR, et al. The global prevalence of hepatitis D virus infection: systematic review and meta-analysis. J Hepatol 2020;73:523–532.
[7] Miao Z, Zhang S, Ou X, et al. Estimating the global prevalence, disease progression, and clinical outcome of hepatitis delta virus infection. J Infect Dis 2020;221:1677–1687.
[8] Meireles LC, Marinho RT, Van Damme P. Three decades of hepatitis B control with vaccination. World J Hepatol 2015;7:2127–2132.
[9] Krsak M, Montague BT, Trowbridge P, et al. Opioid use and chronic infections: the value of addressing the syndemic in correctional settings via telemedicine guidance and broader use of long-acting medications. J Infect Dis 2020;222:S486–S493.
[10] Razavi HA, Buti M, Terrault NA, et al. Hepatitis D double reflex testing of all hepatitis B carriers in low-HBV- and high-HBV/HDV-prevalence countries. J Hepatol 2023;79:576–580.
[11] Observatory Collaborators Polaris. Adjusted estimate of the prevalence of hepatitis delta virus in 25 countries and territories. J Hepatol 2024;80:232–242.
[12] Palom A, Rando-Segura A, Vico J, et al. Implementation of anti-HDV reflex testing among HBsAg-positive individuals increases testing for hepatitis D. JHEP Rep 2022;4:100547.
[13] World Health Organization. Hepatitis B, 2022. https://www.who.int/news-room/fact-sheets/detail/hepatitis-b#:~:text=In%202021%20WHO%20estimated%20that,to%20suppress%20hepatitis%20B%20virus. [Accessed 10 October 2022].
[14] European Association for the Study of the Liver. EASL Clinical Practice Guidelines on hepatitis delta virus. J Hepatol 2023;79:433–460.
[15] Demirel A, Uraz S, Deniz Z, et al. Epidemiology of hepatitis D virus infection in Europe: is it vanishing? J Viral Hepat 2024;31:120–128.
[16] Spain. (2007). Ley 14/2007, de 3 de julio, de investigación biomédica [Law 14/2007, of 3 July, on biomedical research]. Boletín Oficial del Estado, núm. 159, 28826–28848. https://www.boe.es/boe/dias/2007/07/04/pdfs/A28826-28848.pdf; 2007. [Accessed 10 August 2025].
[17] Ordieres C, Navascues CA, Gonzalez-Dieguez ML, et al. Prevalence and epidemiology of hepatitis D among patients with chronic hepatitis B virus infection: a report from Northern Spain. Eur J Gastroenterol Hepatol 2017;29:277–283.
[18] Aguilera A, Trastoy R, Rodriguez-Calvino J, et al. Prevalence and incidence of hepatitis delta in patients with chronic hepatitis B in Spain. Eur J Gastroenterol Hepatol 2018;30:1060–1062.
[19] Gish RG, Jacobson IM, Lim JK, et al. Prevalence and characteristics of hepatitis delta virus infection in patients with hepatitis B in the United States: an analysis of the All-Payer Claims Database. Hepatology 2024;79:1117–1128.
[20] Elsaid MI, Li Y, John T, et al. Economic and health care burdens of hepatitis delta: a study of commercially insured adults in the United States. Hepatology 2020;72:399–411.
[21] Terrault NA, Lok ASF, McMahon BJ, et al. Update on prevention, diagnosis, and treatment of chronic hepatitis B: AASLD 2018 hepatitis B guidance. Hepatology 2018;67:1560–1599.
[22] Buti M, Domínguez-Hernández R, Palom A, et al. Impact of hepatitis D reflex testing on the future disease burden: a modelling analysis. Liver Int 2023;43:2611–2614.
[23] Gish RG, Wong RJ, Di Tanna GL, et al. Association of hepatitis delta virus with liver morbidity and mortality: a systematic literature review and meta-analysis. Hepatology 2024;79:1129–1140.

Keywords: HBV; HDV infection; Clinical burden; Spain.
Received 5 November 2024; received in revised form 21 May 2025; accepted 28 May 2025; Available online 31 May 2025

Journal of Hepatology, Volume *7*

Supplemental information

Clinical burden of HDV in Spain: Incidence, prevalence, and associated comorbidities

Maria Buti, Nandita Kachru, Marvin Rock, Meritxell Ascanio, Josep Darba, and Chong Kim

Clinical burden of HDV in Spain: Incidence, prevalence, and associated comorbidities

Maria Buti, Nandita Kachru, Marvin Rock, Meritxell Ascanio, Josep Darba, Chong Kim

Table of content

Table S1……………………………………………………………………………………….2

Table S1. Baseline Patient Characteristics by Index Diagnosis and Age as a Covariate. All data are presented as n (%) unless stated otherwise. Data were analysed using McNemar test (Chi-squared test). Level of significance set at P < 0.05.

	Pre-2015 Index Diagnosis			Post-2015 Index Diagnosis		
**Comorbidity**	**HDV Infection**	**HBV Monoinfection**	***P*-value**	**HDV Infection**	**HBV Monoinfection**	***P*-value**
**< 45 years**	(n = 83)	(n = 1,294)		(n = 11)	(n = 183)	
Hypertension	2 (2.41)	28 (2.2)	0.4175	1 (9.1)	5 (2.7)	0.3952
History of smoking	18 (21.7)	331 (25.6)	> 0.9999	4 (36.4)	29 (15.9)	0.1857
HCV	7 (8.4)	39 (3.0)	**0.0027**	1 (9.1)	12 (6.6)	0.4236
HIV	11 (13.3)	42 (3.3)	**< 0.0001**	1 (9.1)	6 (3.3)	0.2249
Substance abuse	16 (19.3)	92 (7.1)	**< 0.0001**	2 (18.2)	13 (7.1)	0.3368
AAD/AUD	17 (20.5)	190 (14.5)	0.0982	2 (18.2)	27 (14.8)	0.2413
Diabetes	1 (1.2)	14 (1.1)	0.2764	0	2 (1.1)	> 0.9999
**≥ 45 years**	(n = 48)	(n = 1,149)		(n = 17)	(n = 294)	
Hypertension	1 (2.1)	25 (2.2)	0.4167	1 (5.9)	9 (3.1)	0.4063
History of smoking	11 (22.9)	293 (25.5)	> 0.9999	5 (29.4)	48 (16.3)	0.1725
HCV	4 (8.3)	34 (3.0)	**0.0028**	2 (11.8)	19 (6.5)	0.4297
HIV	7 (14.6)	37 (3.2)	**< 0.0001**	3 (17.7)	10 (3.4)	0.2268
Substance abuse	9 (18.8)	79 (6.9)	**< 0.0001**	3 (17.7)	21 (7.1)	0.3865
AAD/AUD	9 (18.8)	169 (14.7)	0.1085	3 (17.7)	42 (14.3)	0.2741
Diabetes	1 (2.1)	13 (1.1)	0.3751	1 (5.9)	3 (1.0)	> 0.9999

Note: All data are n (%).

AAD/AUD, alcohol abuse disorder/alcohol use disorder; HBV, hepatitis B virus; HCV, hepatitis C virus; HDV, hepatitis delta virus; HIV, human immunodeficiency virus.
